# A Unique Visual Attention Profile Associated With the *FMR1* Premutation

**DOI:** 10.3389/fgene.2021.591211

**Published:** 2021-02-09

**Authors:** Molly Winston, Kritika Nayar, Emily Landau, Nell Maltman, John Sideris, Lili Zhou, Kevin Sharp, Elizabeth Berry-Kravis, Molly Losh

**Affiliations:** ^1^Roxelyn and Richard Pepper Department of Communication Sciences and Disorders, Northwestern University, Evanston, IL, United States; ^2^Chan Division of Occupational Science and Occupational Therapy, University of Southern California, Los Angeles, CA, United States; ^3^Department of Pediatrics, Rush University Medical Center, Chicago, IL, United States

**Keywords:** autism spectrum disorder, social cognition, pragmatic language, eye tracking, fragile X mental retardation gene, fragile X syndrome

## Abstract

Atypical visual attention patterns have been observed among carriers of the fragile X mental retardation gene (*FMR1*) premutation (PM), with some similarities to visual attention patterns observed in autism spectrum disorder (ASD) and among clinically unaffected relatives of individuals with ASD. Patterns of visual attention could constitute biomarkers that can help to inform the neurocognitive profile of the PM, and that potentially span diagnostic boundaries. This study examined patterns of eye movement across an array of fixation measurements from three distinct eye-tracking tasks in order to investigate potentially overlapping profiles of visual attention among PM carriers, ASD parents, and parent controls. Logistic regression analyses were conducted to examine whether variables constituting a PM-specific looking profile were able to effectively predict group membership. Participants included 65PM female carriers, 188 ASD parents, and 84 parent controls. Analyses of fixations across the eye-tracking tasks, and their corresponding areas of interest, revealed a distinct visual attention pattern in carriers of the *FMR1* PM, characterized by increased fixations on the mouth when viewing faces, more intense focus on bodies in socially complex scenes, and decreased fixations on salient characters and faces while narrating a wordless picture book. This set of variables was able to successfully differentiate individuals with the PM from controls (Sensitivity = 0.76, Specificity = 0.85, Accuracy = 0.77) as well as from ASD parents (Sensitivity = 0.70, Specificity = 0.80, Accuracy = 0.72), but did not show a strong distinction between ASD parents and controls (Accuracy = 0.62), indicating that this set of variables comprises a profile that is unique to PM carriers. Regarding predictive power, fixations toward the mouth when viewing faces was able to differentiate PM carriers from both ASD parents and controls, whereas fixations toward other social stimuli did not differentiate PM carriers from ASD parents, highlighting some overlap in visual attention patterns that could point toward shared neurobiological mechanisms. Results demonstrate a profile of visual attention that appears strongly associated with the *FMR1* PM in women, and may constitute a meaningful biomarker.

## Introduction

A range of clinical and subclinical phenotypes result from mutations in the fragile X mental retardation gene (*FMR1*) on the X-chromosome involving a cytosine-guanine-guanine (CGG) repeat expansion in the promotor region of the gene (Bagni and Oostra, 2013). Specifically, a CGG repeat expansion of greater than 200 causes fragile X syndrome (FXS) and inhibits the production of the fragile X mental retardation protein (FMRP), a critical protein involved in synaptic pruning and neural maturation during development (Weiler and Greenough, 1999). FXS is characterized by developmental delays in language, intellectual ability, and social cognition, as well as increased anxiety, and is the leading single-gene disorder associated with autism spectrum disorder (ASD; Bailey et al., 2001; Hernandez et al., 2009). Whereas FXS has a prevalence of around 1 in 4,000 males and 1 in 7,000–10,000 females, the *FMR1* premutation (PM; *FMR1* expansion between 55 and 200 CGG repeats) is more prevalent and occurs in approximately 1 in 468 males and 1 in 151 females in the United States (Seltzer et al., 2012). The *FMR1* PM is also associated with a range of clinical phenotypes, including higher rates of psychiatric and medical comorbidities (Hagerman and Hagerman, 2002; Bourgeois et al., 2011; Besterman et al., 2014).

The PM has unique clinical risks, such as the potential presence of a Parkinson-like neurodegenerative disorder, fragile X-associated tremor and ataxia syndrome (FXTAS), which develops in a subset of carriers with the PM in advanced age (Hagerman et al., 2004). The PM is also associated with a higher risk of psychiatric disorders, including elevated rates of depression and anxiety (Hunter et al., 2008; Bourgeois et al., 2011). Differences in executive skills have also been reported, including working memory, inhibitory control, and processing speed (Wang et al., 2013; Shelton et al., 2014, 2015, 2016). Subclinical phenotypic differences have also been documented in social cognition (Cornish et al., 2005; Losh et al., 2008), language processing (Nayar et al., 2019), and social language use (Losh et al., 2012; Klusek et al., 2016) in women with the PM. Manifestation of the PM phenotype is thought to be in part related to molecular-genetic differences including levels of FMRP and CGG repeat length (Wheeler et al., 2014). Specifically, with respect to this study, some literature suggests that this variation is specifically related to social language (Klusek et al., 2018b) and fixation patterns (Nayar et al., 2019; Winston et al., 2020), although evidence is mixed (Cornish et al., 2005; Wheeler et al., 2014; Klusek et al., 2018a).

There is evidence to suggest that women with the PM may also display differences in patterns of visual fixation, which could reflect underlying differences in social processing and/or cognitive styles, potentially relating to observable clinical-behavioral features. For example, recent work has demonstrated less fluid and coordinated eye-voice control, wherein PM carriers displayed a greater number of repeat fixations during a task of concurrent eye tracking and language processing (Nayar et al., 2019). This less efficient eye-voice coordination not only related to reduced language automaticity and fluency, but also related to greater social language atypicalities and underlying molecular-genetic variation among PM carriers. In another study that examined looking patterns during a face processing task displaying face stimuli exhibiting either direct vs. averted gaze, Klusek et al. (2017) found that women with the PM did not show a preference for either gaze type; in contrast, controls demonstrated a clear attentional preference toward faces showing direct gaze. Importantly, increased dwell time (i.e., time spent fixating on a certain stimulus) on faces exhibiting direct gaze was associated with better social language use in controls but not in the PM, suggesting that PM carriers may not effectively capitalize on information from the eye region of the face. Relatedly, when women with the PM were asked to passively view a series of affective facial expressions, Winston et al. (2020) found that PM carriers displayed an atypical fixation pattern characterized by reduced attention to the eyes and increased attention to the mouth compared to control participants. Interestingly, this atypical fixation pattern was associated with better social language and social cognitive abilities in PM carriers, suggesting that the unique fixation pattern in PM carriers in this task may reflect different strategies for emotion processing than those employed by controls. Together, these results suggest that carriers of the PM demonstrate a different fixation style than controls which may be uniquely modulating social cognition and social language use.

Some studies have also demonstrated phenotypic similarities between carriers of the *FMR1* PM and first-degree relatives of individuals with ASD, who display subtle social cognitive and social language differences as well that are believed to reflect underlying genetic liability to ASD. Specifically, there is evidence that both PM carriers and parents of individuals with ASD show differences from controls on tasks tapping social cognition, such as when asked to infer emotions from the eye region of the face (Cornish et al., 2005; Losh et al., 2009). Moreover, a subgroup of parents of individuals with ASD as well as PM carriers demonstrate a constellation of subclinical traits that parallel the core features of ASD, referred to as the broad autism phenotype (BAP; Losh et al., 2012; Schneider et al., 2016). Indeed, results from Losh et al. (2012) suggest that PM carriers demonstrate elevated levels of personality traits (such as greater social aloofness and rigidity) and social language features consistent with that of the BAP. In addition, higher rates of clinical ASD have also been reported among PM carriers (Clifford et al., 2007).

Individuals with ASD as well as their parents show differences in gaze that complement the patterns observed in the PM. Many studies have shown that individuals with ASD tend to look less at the eyes when exploring faces and more at the mouth (Klin et al., 2002), spend less time looking at social features in scenes (Lee et al., 2019) and demonstrate decrease gaze-language coordination (Nayar et al., 2018) compared to controls. In parents of individuals with ASD, Lee et al. (2019) additionally demonstrated that parents of individuals with ASD who met criteria for the BAP showed distinct gaze profiles when viewing emotionally evocative scenes depicted in the thematic apperception test (TAT), allotting greater attention to faces in scenes in which the faces were featured most prominently, and more attention toward the setting in more complex images with salient setting features. These differences in viewing patterns were associated with the quality of participants’ narratives when telling stories about these scenes (particularly in the group which met criteria for the BAP), suggesting that atypical attentional patterns importantly relate to social communication. Furthermore, Nayar et al. (2018) observed that parents of individuals with ASD, particularly those who met criteria for the BAP, had reduced eye-voice coordination during rapid automatized naming, which was similarly linked with social language abilities, mirroring results reported among female carriers of the *FMR1* PM (Nayar et al., 2019). Together, results demonstrate that parents of individuals with ASD may demonstrate complementary looking patterns to PM carriers, potentially suggesting similar biological underpinnings of these shared phenotypic features.

Given the complex clinical and neurocognitive phenotypes associated with the *FMR1* PM, there is a need for research investigating endophenotypes, or intermediate phenotypes associated with a disorder or condition, but more proximally related to underlying genetics (Gottesman and Gould, 2003). Visual attention profiles may constitute a candidate endophenotype in the PM that overlaps with parents of individuals with ASD. Visual attention is easy to assay and holds strong connections to underlying biology, which may be relatable to downstream clinical-behavioral features that can be used to stratify biologically meaningful subgroups that may cross standard diagnostic boundaries (Frazier et al., 2016, 2018; Pierce et al., 2016). Studying how such potential endophenotypes may overlap in the *FMR1* PM and ASD relatives could offer insight into the potential role of the *FMR1* gene in ASD-related phenotypes. In this way, single-gene disorders such as FXS and others (e.g., 22q11.2 deletion syndrome and Angelman syndrome) have informed understanding of ASD-related phenotypes linked to known genetic variation (Peters et al., 2004; Fine et al., 2005).

A challenge to understanding whether visual attention profiles might constitute such endophenotypes, however, is that most studies of visual attention have focused on single tasks within a participant group, leaving unclear whether more pervasive visual attention profiles might exist across different contexts. It is possible that findings may be task dependent, as seen in other populations (Chawarska et al., 2012), and that important, broader attentional patterns might be revealed by examining performance over multiple tasks and contexts. Therefore, this study aimed to explore patterns of gaze across a series of different eye tracking tasks tapping social attention in complementary ways, including passive viewing of affective facial expressions, tasks requiring narration from a picture book, and a set of emotionally evocative scenes. This study makes use of existing fixation data previously analyzed independently only for group comparisons (Lee et al., 2019; Winston et al., 2020), as well as newly processed data from ASD parents and PM carriers, to build on prior work by applying a more powerful, statistically-driven, cross-contextual approach that combines such existing data within the same participants to characterize more comprehensive fixation profiles that may be characteristic of the PM phenotype, or potentially show overlap with ASD-related profiles. The primary goal of the study was to characterize fixation patterns across tasks that might best predict group membership, and in the case of carriers of the *FMR1* PM, potentially constitute specific gaze profiles associated with *FMR1* as well as identify areas of overlap among groups. We predicted that PM carriers would demonstrate a distinct fixation pattern that could be used to differentiate them from controls with high sensitivity and specificity, but not necessarily from parents of individuals with ASD, due to prior literature suggesting overlapping phenotypic features. Knowledge of such profiles could help to inform the phenotypic expression of the *FMR1* PM, and whether phenotypic overlap exists within related conditions. This information would be informative for stratifying groups based on shared phenotypes, for clinical and research purposes.

## Materials and Methods

### Participants

Participants included 65 adult females with the *FMR1* PM (PM group), 188 parents of individuals with ASD (ASD parent group), and 84 parent controls (control group). As noted previously, a subset of participants was included in two previous reports examining the eye tracking tasks in isolation (Lee et al., 2019; Winston et al., 2020). New data are included as well, consisting of fixations to the facial stimuli task for ASD parents (whose fixation data to the narrative tasks were included in Lee et al., 2019) and fixation data from the two narrative tasks for PM carriers (whose face fixation data were reported previously in Winston et al., 2020). The aims, analyses, and results addressed in the present study are unique and have not been previously examined. The previously published research and rich existing dataset available for this study permitted a statistically-driven approach to investigate fixations across different social-emotional stimuli that might reveal visual fixation profiles that serve as endophenotypic markers. Control families were screened for personal or family history of ASD, FXS, or related neurodevelopmental disorders, including language-related delays. Carrier status was confirmed through analysis of CGG repeats either within the experimental protocol or confirmation from medical records. All participants were under the age of 66. Participants with the *FMR1* PM were additionally screened for FXTAS symptoms. Participants were also excluded if their Full Scale IQ was below 80 [obtained using the Wechsler Abbreviated Scale of Intelligence (WASI; Wechsler, 1999)]. The WASI was administered to all participants by a trained examiner and conducted in the laboratory, a quiet space in the participant’s home, or another controlled environment that was convenient for participants if travel to the lab was not possible. The procedures for task administration were consistent across all tasks and groups, as described in Winston et al. (2020) and Lee et al. (2019). Significant differences between groups emerged for age (*p* = 0.006) and Full Scale IQ (*p* = 0.02); however, age was not associated with outcome variables (*ps* > 0.26), and IQ was only associated with one outcome variable (looking time toward faces; *p* = 0.02), and so were not included in subsequent analyses (see Table 1). Additionally, because this study investigated the utility of eye tracking to classify groups, it was important to allow for full phenotypic variability, which may include differences in cognition. Sex differences within the control group and ASD parent group were assessed for the primary outcome variables using independent samples *t*-tests. No significant differences were observed (*ps* > 0.18), so the male and female groups were combined for data analyses. The study protocol was approved by Northwestern University’s Institutional Review Board.

**Table 1:** Ratio of sex distribution, mean (M), and standard deviation (SD) for chronological age and IQ across groups.

	Control group	ASD parent group	PM group
	*M* (*SD*)	*M* (*SD*)	*M* (*SD*)
Males:Females	31:53	69:119	0:64
Chronological age	41.84 (10.11)	46.09 (7.86)	45.28 (10.17)
Full-scale IQ	116.85 (10.26)	111.67 (11.65)	111.84 (9.94)
CGG repeats	--	30.41 (4.23)	88.76 (16.64)
Quantitative FMRP	--	0.02 (0.008)	0.02 (0.008)
Activation ratio	--	--	0.47 (0.24)

M = mean; SD = standard deviation.

### Eye-Tracking Stimuli

#### Wordless Picture Book

Participants viewed a 24-page wordless picture book, *Frog Where Are You?* (Mayer, 1969), about a boy and his dog searching for their lost pet frog. This picture book has been used frequently in studies of narrative across populations, and recently, to study visual attention during narration (Thurber and Tager-Flusberg, 1993; Tager-Flusberg, 1995; Losh and Capps, 2003; Norbury and Bishop, 2003; Reilly et al., 2004; Diehl et al., 2006; Colle et al., 2008; Sah and Torng, 2015). Participants narrated the story as they simultaneously viewed each page of the picture book on an eye-tracker. There was no time limit for completing the task. Administration methods were consistent with previous studies using this picture book task.

#### Thematic Apperception Test

Participants were shown a subset of six images varying in complexity from the TAT (Murray, 1943), a projective psychological test that uses ambiguous images. The TAT has been used in previous studies to elicit narratives, and the six images were selected to align with previous work (Lee et al., 2019). Participants viewed each image for 8s and then were asked to tell a story about the image with a beginning, middle, and end and with details about the character’s thoughts, feelings, and actions.

#### Affective Facial Expressions

Images from the NimStim Facial Stimulus Set (Farzin et al., 2009; Tottenham et al., 2009) were presented to participants and depicted happy, calm, and fearful faces. There were a total of 60 faces, comprised of 20 happy, 20 calm, and 20 fearful. In accordance with procedures outlined in Winston et al. (2020), participants first viewed a gray screen, then a scrambled face controlling for luminance for 1s, and then a face depicting an emotion for 3s.

### Data Processing

All tasks were presented to participants on a Tobii T60 eye tracker and gaze data were recorded from both eyes. For the picture book task, areas of interest (AOIs) were established including animate (i.e., all characters), protagonist (i.e., boy, dog, and frog), inanimate (i.e., setting), and the protagonist’s focus of attention (i.e., where the protagonist is looking) regions. For the TAT task, AOIs included animate, face, body, and inanimate regions. AOIs from the TAT were generated for each image separately which corresponded to a category based on the predominant AOIs (setting, bodies, and faces). AOIs were expanded by 10% to account for loss of tracked fixations. For the TAT task, data for an image were excluded if there was less than 4s of tracked fixations (out of a possible 8s maximum). See (Lee et al., 2019) for a detailed explanation of eye tracking and quality control procedures for the picture book and TAT stimuli.

For the NimStim Facial Stimulus Set (AFE task), AOIs were established for the eyes, nose, and mouth regions of each face, and fixations to AOIs were analyzed by the proportion fixation duration to AOIs. Images were excluded if there were less than 50% of fixations for each image and if 50% of all trials were poor quality. See Winston et al. (2020) for an in-depth explanation of eye tracking and quality control procedures for pupil diameter and gaze fixation data.

### Clinical-Behavioral Characteristics

#### Social Cognition

Participants completed the Reading the Mind from the Eyes Task (Baron-Cohen et al., 2001) where they were required to infer complex psychological expressions from a pair of eyes. There were 36 images of eyes. Scores were determined by the proportion of correct responses out of 36 (i.e., the maximum number of potential correct responses).

#### Social Language

The Pragmatic Rating Scale (PRS; Landa et al., 1992) was used to assess social language. Participants engaged in a semi-structured conversation about their life experiences, and the interactions were coded by raters blind to diagnosis status for pragmatic language violations (e.g., excessive detail and informal language).

#### Personality Features

Personality features related to the BAP (e.g., aloofness and rigidity) was assessed using the self-report version of the Broad Autism Phenotype Questionnaire (BAPQ; Hurley et al., 2007). The BAPQ consists of 36 items which were rated on a 6-point Likert scale ranging from “very rarely” to “very often.”

### *FMR1* Molecular Characterization

Blood samples were collected from 49PM carriers and processed to determine CGG repeat length, quantitative FMRP (pg/ug), and activation ratio (i.e., the percentage of cells which contain an X chromosome with an unaffected *FMR1* gene). FMRP was derived using the Luminex Technology immunoassay whereas *FMR1* genotyping was done with a highly sensitive PCR method (Asuragen; Filipovic-Sadic et al., 2010; LaFauci et al., 2013), and activation ratio was determined by a Southern blot protocol (Berry-Kravis et al., 2005; LaFauci et al., 2013). CGG repeat length and FMRP were also ascertained on 108 ASD parents using the aforementioned protocols.

### Data Analysis Plan

#### Variable Selection

To determine the set variables across tasks which could be used as predictors of PM status, potentially constituting a PM-specific visual attention profile, group differences between individuals with the PM and controls were explored across the primary variables in each eye-tracking task using independent samples *t*-tests. One variable per task or per image if indicated for the TAT was then selected for subsequent profile analyses based on significance and effect size. Variability was also examined using normality statistics to assess for possible reasons for subsequent findings of measurement variance. Investigating across AOIs derived from the PB, PM carriers differed most from controls in their looking time toward the protagonist’s focus of attention (PB-FOA; *t* = 3.20, *p* = 0.002, *d* = 0.53). Within the TAT, in the setting based slides, the PM carriers spent less time than controls looking at faces in one of two setting-dominated scenes depicting a surgical procedure (TAT Setting-Faces; *t* = 2.15, *p* = 0.03, *d* = 0.35) as well as in one of two body-dominant scenes (TAT Bodies-Faces; *t* = 2.07, *p* = 0.04, *d* = 0.33). Furthermore, within the TAT, PM carriers spent more time fixating on bodies in one of two face-dominant scenes (TAT Faces-Bodies; *t* = −1.89, *p* = 0.03, *d* = 0.31). As noted previously, findings related to differences between the ASD parent and control group in the context of the PB and TAT have been previously published (Lee et al., 2019). Finally, across all faces employed in the passive viewing task of affective facial expressions, PM carriers spent significantly more time fixating on the mouth compared to controls (AFE-Mouth; *t* = −39.95, *p* < 0.001, *d* = −6.57). Results related to this difference have been reported in greater detail in Winston et al. (2020).

#### Logistic Regression

Logistic regressions were conducted to assess the utility of a set of variables in which the PM carriers differed from controls as predictors of group membership. For logistic regressions, variables in which the PM group differed from controls were entered into the model (i.e., PB-FOA, TAT Setting-Faces, TAT Bodies-Faces, TAT Faces-Bodies, and AFE-Mouth), and predictive power was assessed. The mice package in *R Studio* was used for multiple imputation using chained equations with predictive mean matching to address missingness across tasks (van Buuren and Groothuis-Oudshoorn, 2011) using 70 imputed datasets using calculations based on missingness detailed in von Hippel (2020). Overall, 30% of the observations was imputed, 10% from the PB, 32% from TAT, and 70% from AFE. This imputation method takes into account all relevant fixation variables as well as group status to make predictions for missing values allowing for robust predictions despite great degrees of missingness (Vink et al., 2014). Pooled parameter results were obtained including pooled r^2^, area under the curve (AUC; broadly denoting accuracy), and coefficients with the psfmi package. Variable selection was conducted using the backward stepwise selection and the pooling sampling variance method using a p-criterion of 0.10 to allow for a more exploratory approach.

Additional analyses of specificity and sensitivity were conducted on the stacked dataset, which has been shown to yield comparable results to more complex methodology (Thao and Geskus, 2019). A series of pairwise logistic regression analyses were conducted to assess the variables’ predictive power and discriminability between the PM and control groups, PM and ASD parent group, and ASD parent and control group.

#### Correlations With *FMR1*-Related Variation

Pearson correlations were conducted to investigate relationships between key variables of interest and *FMR1* molecular-genetic variation in the PM group and ASD parent group.

#### Secondary Analysis of Group Classification Results

Using Mann-Whitney *U* tests to account for unequal sample sizes, we examined whether PM carriers who were misclassified in the logistic regression model as ASD parents might display higher rates of ASD-related features that comprise the BAP (e.g., pragmatic language differences), and may also show differences in *FMR1*-related molecular-genetic variability. Imputed values were averaged for these exploratory analyses due to constraints of the use of pooled imputed datasets. We also explored whether children with FXS of the misclassified PM parents may exhibit differences in ASD symptoms from children of correctly classified parents, by examining ASD symptom severity on the Autism Diagnostic Observation Schedule-2 (ADOS-2; Lord et al., 2001; Gotham et al., 2008).

## Results

### Logistic Regression

#### PM Group vs. Control Group

Results indicate that PB-FOA and AFE-Mouth were all significant predictors of group status independently (*ps* < 0.05), with an overall pooled *r*^2^ = 0.30 and AUC of 0.78 (maximum specificity = 0.71 and sensitivity = 0.68). The simplest model maintaining predictive power included only the PB-FOA and AFE-Mouth, with a pooled ROC of 0.77 (maximum specificity = 0.85 and sensitivity = 0.76). This model, conducted in the averaged imputed dataset, correctly classified 92% of the participants (77/84) from the control group and 68% PM carriers (44/65).

#### PM Group vs. ASD Parent Group

With each variable comprising the composite entered in the logistic regression model AFE-Mouth was the only independent significant predictor of group status (*p* < 0.001). Overall, the model incorporating all variables was able to predict group status with a pooled AUC of 0.74 (maximum specificity = 0.71, sensitivity = 0.68). The stepwise logistic regression model only maintained AFE-Mouth with a pooled AUC of 0.72 (maximum specificity = 0.80 and sensitivity = 0.70). This model, conducted in the average imputed dataset, correctly classified 98% of the ASD parents (183/188) and 62% of the PM carriers (40/65).

#### ASD Parent Group vs. Control Group

Of all the variables included in the model, only PB-FOA was a significant predictor of group status (*p* = 0.05). Together, the model with all variables had a pooled AUC of 0.62 (*r*^2^ = 0.05; maximum specificity = 0.59 and sensitivity = 0.56). The stepwise regression model only maintained PB-FOA and comparable accuracy (0.60), specificity (0.52), and sensitivity (0.62). The final model correctly classified 98% of the ASD parents (185/188) and 4% of the control participants (3/84).

**Figure 1:**
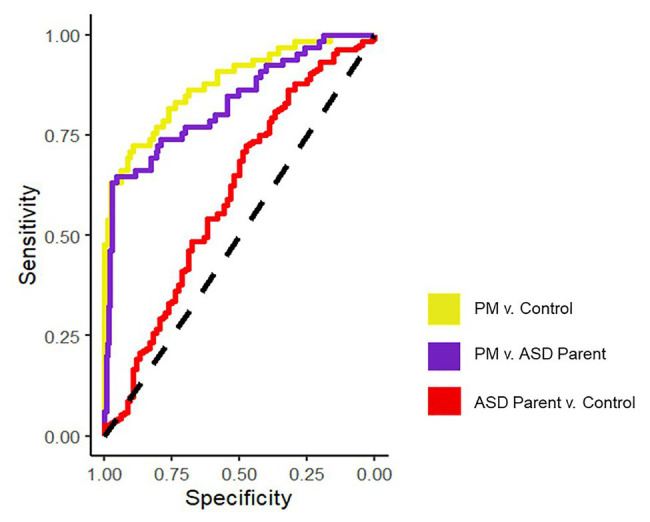
ROC curves assessing discriminability across groups for the final logistic regression models on the averaged datasets.

See Figure 1 for all pairwise ROC curves. See Table 2 for model parameters for each logistic regression model.

**Table 2:** Model parameters for each logistic regression model.

	Predictor	Original model			Final model		
		Estimate	Confidence interval	*p*	Estimate	Confidence interval	*p*
***PM vs*. *TD***							
TAT	Setting: Faces^3^	−0.01	−0.05, 0.03	0.56	--	--	--
	Bodies: Faces^1^	−0.002	−0.03, 0.03	0.89	--	--	--
	Faces: Bodies^2^	0.004	−0.04, 0.04	0.85	--	--	--
Picture book	Focus of attention	−0.13	−0.26, −0.003	0.05	−0.15	−0.27, −0.04	0.008
Affective facial expressions	Mouth	0.03	0.01, 0.05	0.001	0.03	0.01, 0.05	<0.001
***PM vs*. *ASD parents***							
TAT	Setting: Faces^4^	−0.02	−0.04, 0.01	0.20	--	--	--
	Bodies: Faces^2^	0.01	−0.01, 0.03	0.49	--	--	--
	Faces: Bodies^1^	0.005	−0.02, 0.03	0.67	--	--	--
Picture book	Focus of attention^3^	−0.06	−0.17, 0.05	0.29	--	--	--
Affective facial expressions	Mouth	0.03	0.01, 0.05	<0.001	0.03	0.01, 0.04	<0.001
***ASD parents vs*. *TD***							
TAT	Setting: Faces^3^	0.002	−0.02, 0.02	0.83	--	--	--
	Bodies: Faces^1^	−0.01	−0.03, 0.01	0.36	--	--	--
	Faces: Bodies^4^	0.0002	−0.02, 0.02	0.99	--	--	--
Picture book	Focus of attention	−0.09	−0.18, −0.01	0.04	−0.11	−0.19, −0.02	0.02
Affective facial expressions	Mouth^2^	0.0006	−0.01, 0.01	0.91	--	--	--

Superscript denotes when the variable was excluded in the stepwise logistic regression procedure.

### Correlations With *FMR1*-Related Variation

**Figure 2:**
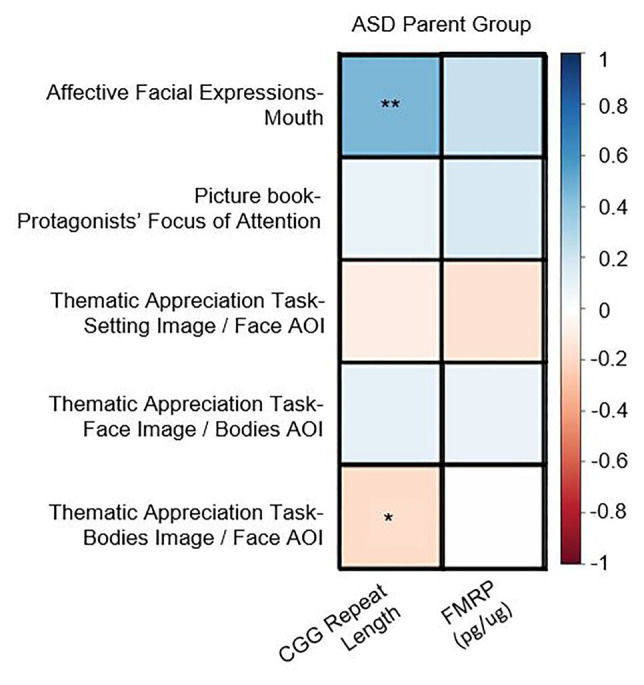
Correlation matrix for genetics associations observed in the autism spectrum disorder (ASD) parent group across key areas of interest (AOI; ^*^p < 0.05, ^**^p < 0.01).

In the PM group, there were no significant correlations between CGG repeat length, FMRP, or activation ratio (*ps* > 0.13) and any of the eye-tracking variables [*note*: a significant correlation between CGG repeats and AFE-mouth was similarly not observed in Winston et al. (2020)]. In the ASD parent group, increased CGG repeats were associated with increased time spent looking at the mouth in the AFE task (*r* = 0.45, *p* = 0.005) as well as decreased time spent looking at the face in the TAT (*r* = –0.22, *p* = 0.037; see Figure 2). No associations were observed with FMRP in the ASD parent group (*ps* > 0.13).

### Secondary Analyses of Misclassified PM Carriers

**Figure 3:**
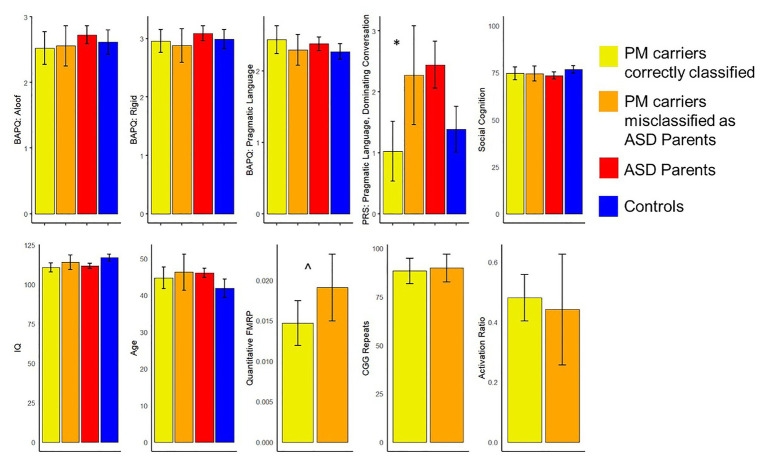
Comparisons of clinical-behavioral and *FMR1*-related variables between the premutation (PM) carriers who were misclassified as ASD parents and those who were correctly classified (^*^p < 0.05, ^^^*p* < 0.10). Of note, controls are not included in comparisons related to broad autism phenotype (BAP) features, and ASD parents and controls were not included in comparisons related to *FMR1* molecular-genetic variation.

Exploratory analyses investigated whether those PM carriers who were misclassified as ASD parents (*n* = 25) might differ from correctly classified PM carriers along any clinical-behavioral characteristics. Results indicated that PM carriers who were incorrectly identified as ASD parents demonstrated greater pragmatic language violations as reflected by the dominating conversation factor (e.g., overly talkative and tangential; *U* = 244.50, *p* = 0.006) and marginally greater FMRP than the correctly classified carriers (*U* = 155.00, *p* = 0.08). They did not differ on any other features, including personality features of the BAP, social cognition, IQ or age (*ps* > 0.17). See Figure 3 for all group comparisons.

**Figure 4:**
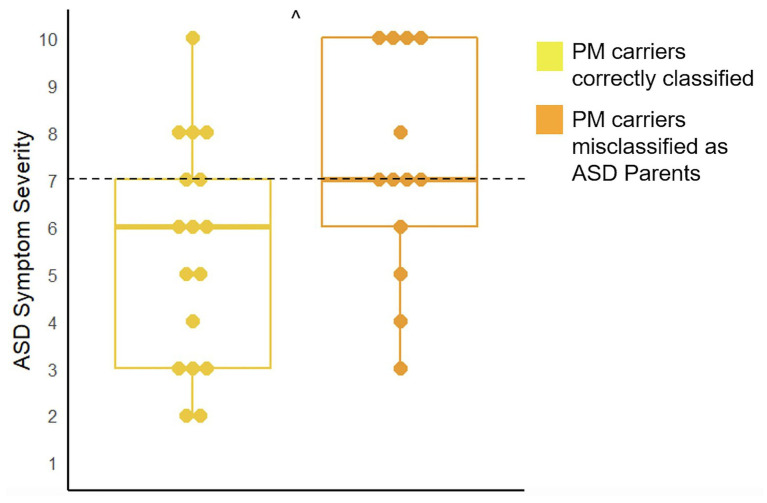
Comparison of ASD symptom severity as measured by the ADOS-2 in the children of PM carriers who were misclassified as ASD parents and those who were correctly classified (^^^*p* < 0.10). The cut-off for meeting criteria for ASD is seven (range: 1–10).

There were 13 children included in analyses for the 25PM carriers who were misclassified as ASD parents and 17 children included in analyses for the 40 correctly classified PM carriers. Results demonstrated that children of the misclassified PM carriers had marginally higher ASD symptom severity (*U* = 67.00, *p* = 0.07) and displayed more restrictive and repetitive behaviors (*U* = 63.50, *p* = 0.05; See Figure 4).

## Discussion

The present study examined patterns of looking across three separate eye tracking tasks in female carriers of the *FMR1* PM, parents of individuals with ASD, and controls, using a series *t*-tests and logistic regression analyses to investigate whether there may exist patterns of visual fixations across eye-tracking tasks, representing a PM-specific profile, that could effectively predict group membership. Results demonstrated that female carriers of the *FMR1* PM showed a distinct looking pattern in the affective facial expressions task, but across the other two tasks (i.e., a wordless picture book and images from the thematic apperception task), exhibited fixation patterns that were similar to ASD parents. Analysis of group classification revealed that this looking pattern may also be used to further differentiate a subgroup of PM carriers who display some degree of subclinical ASD-related features.

The PM Profile was comprised of variables across each of the three eye-tracking tasks in which PM carriers differed from controls with the highest effect size, including (1) time spent fixating on the protagonists’ focus of attention in the wordless picture book (PB), (2) time spent fixating on faces and bodies in complex social scenes (TAT), and (3) time spent fixating on the mouth in the NimStim passive viewing task of affective facial expressions (AFE). Logistic regression analyses revealed differences in predictive power across these variables, highlighting the importance of fixations towards the mouth in particular in differentiating PM carriers from both ASD parents and controls. This finding is consistent with previous research suggesting that individuals with the *FMR1* PM attend to different features of the face (i.e., the mouth), whereas controls typically fixate on the eye region of the face (Bentin et al., 1996; Farroni et al., 2002; Henderson et al., 2005). Interestingly, in prior work, parents of individuals with ASD showed greater reliance on the mouth than other areas of the face when making affective judgments (Adolphs et al., 2008), and differences in facial processing have also been noted (Losh et al., 2009), and linked to underlying neural correlates such as amygdala activation (Yucel et al., 2015), similar to findings from studies of PM males (Hessl et al., 2007). However, results from the present study highlight looking towards the mouth as a more specific predictor of PM status, despite some differences present in ASD parents as well. A prior study including a subset of the present sample demonstrated that increased time spent fixating on the mouth was associated with better social cognitive abilities and fewer pragmatic (i.e., social) language violations (Winston et al., 2020). Likewise, Klusek et al. (2017) showed that PM carriers did not demonstrate a preference towards direct gaze compared to averted gaze, and there was no relationship between time spent fixating on the eyes and better pragmatic language abilities, in contrast to results observed in the control group. Taken together with prior work, these findings might suggest that PM carriers use different visual processing strategies which may affect social engagement.

The other variables which comprise the PM Profile were not statistically selected in analyses using backward selection for the PM and ASD parent groups and are particularly noteworthy given prior evidence of phenotypic similarities between these groups, including pragmatic language, social cognition, and personality features consistent with the BAP (Losh et al., 2012; Klusek et al., 2017). When examining social scenes, parents of individuals with ASD included in the present study, particularly those exhibiting features of the BAP, spent more time fixating on the background of complex social scenes in the TAT (Lee et al., 2019), which is consistent with patterns observed in the PM in which they spent less time fixating on emotionally salient aspects of scenes (e.g., faces). This similarity in looking patterns may account for the results here indicating that individual fixations within scenes in the TAT and PB were unable to differentiate group the PM group from the ASD parent group with sufficient sensitivity and specificity. Therefore, while these particular AOIs may not be informative in differentiating the PM group from the ASD parent group, they may be useful in tapping similarities in neurobiology or genetics underlying attention allocation in complex social scenes. In contrast, exhibiting increased fixations toward the mouth during face processing tasks might reflect a more specific characteristic of the PM group.

Interestingly, secondary analyses examining phenotypic differences in PM carriers who were incorrectly classified as ASD parents showed that this group demonstrated increased pragmatic language violations (at rates similar to the ASD parent group), and higher levels of FMRP, but did not differ in IQ, age, social cognition, or other BAP features. Furthermore, children of the group of PM carriers who were misclassified as ASD parent’s demonstrated overall greater ASD symptom severity, and more severe repetitive behaviors than children from the group of correctly classified as PM carriers. Evidence that higher rates of ASD-related characteristics were present in the misclassified PM carriers and in their children with FXS could suggest the presence of additional etiologic factors in this subgroup of families, such as differences in genetic background, including variation in ASD risk genes known to interact with *FMR1* (Belmonte and Bourgeron, 2006; Darnell et al., 2011; Hagerman et al., 2011; Steinberg and Webber, 2013). As such, these findings may warrant further investigation into the specific phenotypes (and aspects of pragmatic language in particular) that might distinguish a cluster of PM carriers who share features with ASD relatives. Such overlapping phenotypes could help to guide investigations into etiologic factors related to ASD features in *FMR1* mutation conditions (e.g., potential involvement of ASD risk genes that are known interactors with *FMR1*).

Correlational analyses revealed an association between higher CGG repeats and time spent looking at the mouth on the affective facial expressions task specifically in the ASD parent group. This association highlights that *FMR1* CGG repeats within the normal range may be related to PM-specific traits. Higher CGG repeats within the PM range have been associated with a range of PM phenotypic characteristics (Wheeler et al., 2014), and this finding adds to some literature suggesting that variation within the gray zone or at the upper/lower end of the normal range may confer risk for medical problems and cognitive differences (Bretherick et al., 2005; Mailick et al., 2014). Findings reported here suggest that visual processing strategies may be an additional such phenotype, influenced by even subtle *FMR1* variation.

Taken together, results of this study highlight the utility of eye tracking in the context of a face processing task as a tool for prediction of PM status and specific endophenotypic marker. Findings also provide evidence of overlap in visual fixation patterns with parents of individuals with ASD, suggesting potentially shared neurobiological mechanisms underlying phenotypes observed across these populations. Results may therefore help to elucidate etiology for ASD traits that may express across different diagnostic boundaries. Likewise, these indices reflecting shared clinical-behavioral features may be used to further stratify the PM group into more clinically, and potentially etiologically homogenous subgroups for use in clinical and biological studies. Furthermore, results support the utility of eye-tracking data to characterize endophenotypic markers in female carriers of the *FMR1* PM, and illustrate the unique predictive power of specific variables derived using eye-tracking data in differentiating group membership.

Several strengths and limitations of the study should be considered in the interpretation of results. A strength of this study includes the data-driven investigation of visual attention profiles across contexts and multiple groups of relatively substantial sample size. Importantly, by moving beyond investigation of fixation data within single tasks, analyses aimed to characterize broader visual attention profiles that may characterize specific groups, potentially show group overlap, and help to target studies connecting these phenotypes to underlying biology. Studying visual attention across tasks that varied in complexity and social demands permitted a relatively comprehensive assessment of attentional patterns that might contribute to understanding of the *FMR1* PM phenotype (Klin et al., 2002; Shic et al., 2011; Chawarska et al., 2012). Additionally, the inclusion of parents of individuals with ASD as an additional comparison group with carriers of the *FMR1* PM is important, given the strong overlap between ASD and *FMR1*-related conditions (Cornish et al., 2005; Clifford et al., 2007; Losh et al., 2008; Klusek et al., 2017; Nayar et al., 2018, 2019), and presence of a relatively large number of high confidence ASD risk genes that are known interactors with *FMR1* (Belmonte and Bourgeron, 2006; Darnell et al., 2011; Hagerman et al., 2011; Ascano et al., 2012; Steinberg and Webber, 2013; De Rubeis et al., 2014). Phenotypic comparisons across these groups may help to identify shared endophenotypes that can elucidate common etiologic factors across conditions. A limitation, noted previously, is that not all participants contributed data for each task. However, mitigating this concern is that analyses applied multiple imputation using chained equations (MICE) with predictive mean matching, and auxiliary variables to inform imputation and avoid biases present in other single imputation techniques (Schafer, 1999; Stuart et al., 2009; Azur et al., 2011). Future work with larger samples would benefit from split datasets, to permit training and testing, and may also fruitfully consider machine learning approaches and cross-validation measures. Finally, it will be important to investigate whether the PM visual attention profile identified here might extend to males with the PM, and constitute a *FMR1*-related endophenotype that could help to characterize the profile of the *FMR1* PM in males and females, and guide future investigations of gene-brain-behavior connections in PM carriers.

## Data Availability Statement

The datasets presented in this study can be found in online repositories. The names of the repository/repositories and accession number(s) can be found at: https://nda.nih.gov/edit_collection.html?id=1958.

## Ethics Statement

The studies involving human participants were reviewed and approved by Northwestern University Institutional Review Board. The patients/participants provided their written informed consent to participate in this study.

## Author Contributions

MW conceptualized project questions, analyzed the data, and drafted the initial manuscript. KN, EL, and NM assisted in preparing the dataset and provided valuable feedback on manuscript drafts. KN and EL further assisted in completing verifications of published results as well as contributed to manuscript preparation. JS provided further consultation and verification of statistical methods and results. LZ, KS, and EB-K provided resources for genetic analysis of the *FMR1* gene, helped to draft the Materials and Methods section pertaining to genetic analyses, and provided essential expertise in the interpretation of results. ML secured funding for the projects from which these data were drawn, helped to conceptualize the project, interpret data, and develop the manuscript. All authors contributed to the article and approved the submitted version.

### Conflict of Interest

The authors declare that the research was conducted in the absence of any commercial or financial relationships that could be construed as a potential conflict of interest.

## References

[ref1] Adolphs R. Spezio M. L. Parlier M. Piven J. (2008). Distinct face-processing strategies in parents of autistic children. Curr. Biol. 18, 1090–1093. 10.1016/j.cub.2008.06.073 18635351 PMC2504759

[ref2] Ascano M. J. Mukherjee N. Bandaru P. Miller J. B. Nusbaum J. D. Corcoran D. L.. (2012). FMRP targets distinct mRNA sequence elements to regulate protein expression. Nature 492, 382–386. 10.1038/nature11737, PMID: 23235829 PMC3528815

[ref3] Azur M. J. Stuart E. A. Frangakis C. Leaf P. J. (2011). Multiple imputation by chained equations: what is it and how does it work? Int. J. Methods Psychiatr. Res. 20, 40–49. 10.1002/mpr.329 21499542 PMC3074241

[ref4] Bagni C. Oostra B. A. (2013). Fragile X syndrome: from protein function to therapy. Am. J. Med. Genet. A 161A, 2809–2821. 10.1002/ajmg.a.36241, PMID: 24115651

[ref5] Bailey D. B. J. Hatton D. D. Skinner M. Mesibov G. (2001). Autistic behavior, FMR1 protein, and developmental trajectories in young males with fragile X syndrome. J. Autism Dev. Disord. 31, 165–174. 10.1023/a:1010747131386, PMID: 11450815

[ref6] Baron-Cohen S. Wheelwright S. Hill J. Raste Y. Plumb I. (2001). The "Reading the Mind in the eyes" test revised version: a study with normal adults, and adults with Asperger syndrome or high-functioning autism. J. Child Psychol. Psychiatry 42, 241–251. 10.1111/1469-7610.00715, PMID: 11280420

[ref7] Belmonte M. K. Bourgeron T. (2006). Fragile X syndrome and autism at the intersection of genetic and neural networks. Nat. Neurosci. 9, 1221–1225. 10.1038/nn1765, PMID: 17001341

[ref8] Bentin S. Allison T. Puce A. Perez E. McCarthy G. (1996). Electrophysiological studies of face perception in humans. J. Cogn. Neurosci. 8, 551–565. 10.1162/jocn.1996.8.6.551, PMID: 20740065 PMC2927138

[ref9] Berry-Kravis E. Potanos K. Weinberg D. Zhou L. Goetz C. G. (2005). Fragile X-associated tremor/ataxia syndrome in sisters related to X-inactivation. Ann. Neurol. 57, 144–147. 10.1002/ana.20360, PMID: 15622531

[ref10] Besterman A. D. Wilke S. A. Mulligan T. E. Allison S. C. Hagerman R. Seritan A. L. (2014). Towards an understanding of neuropsychiatric manifestations in fragile X premutation carriers. Future Neurol. 9, 227–239. 10.2217/fnl.14.11. 25013385 PMC4086747

[ref11] Bourgeois J. A. Seritan A. L. Casillas E. M. Hessl D. Schneider A. Yang Y. (2011). Lifetime prevalence of mood and anxiety disorders in fragile X premutation carriers. J. Clin. Psychiatry 72, 175–182. 10.4088/JCP.09m05407blu. 20816038 PMC4038118

[ref12] Bretherick K. L. Fluker M. R. Robinson W. P. (2005). FMR1 repeat sizes in the gray zone and high end of the normal range are associated with premature ovarian failure. Hum. Genet. 117, 376–382. 10.1007/s00439-005-1326-8 16078053

[ref13] Chawarska K. Macari S. Shic F. (2012). Context modulates attention to social scenes in toddlers with autism. J. Child Psychol. Psychiatry 53, 903–913. 10.1111/j.1469-7610.2012.02538.x 22428993 PMC3845814

[ref14] Clifford S. Dissanayake C. Bui Q. M. Huggins R. Taylor A. K. Loesch D. Z. (2007). Autism spectrum phenotype in males and females with fragile X full mutation and premutation. J. Autism Dev. Disord. 37, 738–747. 10.1007/s10803-006-0205-z, PMID: 17031449

[ref15] Colle L. Baron-Cohen S. Wheelwright S. van der Lely H. K. (2008). Narrative discourse in adults with high-functioning autism or Asperger syndrome. J. Autism Dev. Disord. 38, 28–40. 10.1007/s10803-007-0357-5, PMID: 17345168

[ref16] Cornish K. Kogan C. Turk J. Manly T. James N. Mills A.. (2005). The emerging fragile X premutation phenotype: evidence from the domain of social cognition. Brain Cogn. 57, 53–60. 10.1016/j.bandc.2004.08.020, PMID: 15629215

[ref17] Darnell J. C. Van Driesche S. J. Zhang C. Hung K. Y. Mele A. Fraser C. E.. (2011). FMRP stalls ribosomal translocation on mRNAs linked to synaptic function and autism. Cell 146, 247–261. 10.1016/j.cell.2011.06.013, PMID: 21784246 PMC3232425

[ref18] De Rubeis S. He X. Goldberg A. P. Poultney C. S. Samocha K. Cicek A. E.. (2014). Synaptic, transcriptional and chromatin genes disrupted in autism. Nature 515, 209–215. 10.1038/nature13772, PMID: 25363760 PMC4402723

[ref19] Diehl J. J. Bennetto L. Young E. C. (2006). Story recall and narrative coherence of high-functioning children with autism spectrum disorders. J. Abnorm. Child Psychol. 34, 87–102. 10.1007/s10802-005-9003-x, PMID: 16485176

[ref20] Farroni T. Csibra G. Simion F. Johnson M. H. (2002). Eye contact detection in humans from birth. Proc. Natl. Acad. Sci. U. S. A. 99, 9602–9605. 10.1073/pnas.152159999, PMID: 12082186 PMC123187

[ref21] Farzin F. Rivera S. M. Hessl D. (2009). Brief report: visual processing of faces in individuals with fragile X syndrome: an eye tracking study. J. Autism Dev. Disord. 39, 946–952. 10.1007/s10803-009-0744-1, PMID: 19399604 PMC2684976

[ref22] Filipovic-Sadic S. Sah S. Chen L. Krosting J. Sekinger E. Zhang W.. (2010). A novel FMR1 PCR method for the routine detection of low abundance expanded alleles and full mutations in fragile X syndrome. Clin. Chem. 56, 399–408. 10.1373/clinchem.2009.136101, PMID: 20056738 PMC4031651

[ref23] Fine S. E. Weissman A. Gerdes M. Pinto-Martin J. Zackai E. H. McDonald-McGinn D. M.. (2005). Autism spectrum disorders and symptoms in children with molecularly confirmed 22q11. 2 deletion syndrome. J. Autism Dev. Disord. 35, 461–470. 10.1007/s10803-005-5036-9, PMID: 16134031 PMC2814423

[ref78] Frazier T. W. Klingemier E. W. Beukemann M. Speer L. Markowitz L. Parikh S.. (2016). Development of an objective autism risk index using remote eye tracking. J. Am. Acad. Child. Adolesc. Psychiatry 55, 301–309. 10.1016/j.jaac.2016.01.011, PMID: 27015721 PMC4808563

[ref79] Frazier T. W. Klingemier E. W. Parikh S. Speer L. Strauss M. S. Eng C.. (2018). Development and validation of objective and quantitative eye tracking-based measures of autism risk and symptom levels. J. Am. Acad. Child. Adolesc. Psychiatry 57, 858–866. 10.1016/j.jaac.2018.06.023, PMID: 30392627 PMC6220711

[ref24] Gotham K. Risi S. Dawson G. Tager-Flusberg H. Joseph R. Carter A.. (2008). A replication of the autism diagnostic observation schedule (ADOS) revised algorithms. J. Am. Acad. Child Adolesc. Psychiatry 47, 642–651. 10.1097/CHI.0b013e31816bffb7, PMID: 18434924 PMC3057666

[ref25] Gottesman I. I. Gould T. D. (2003). The endophenotype concept in psychiatry: etymology and strategic intentions. Am. J. Psychiatry 160, 636–645. 10.1176/appi.ajp.160.4.636, PMID: 12668349

[ref26] Hagerman R. Au J. Hagerman P. (2011). FMR1 premutation and full mutation molecular mechanisms related to autism. J. Neurodev. Disord. 3, 211–224. 10.1007/s11689-011-9084-5, PMID: 21617890 PMC3261276

[ref27] Hagerman R. J. Hagerman P. J. (2002). The fragile X premutation: into the phenotypic fold. Curr. Opin. Genet. Dev. 12, 278–283. 10.1016/S0959-437X(02)00299-X, PMID: 12076670

[ref28] Hagerman R. J. Leavitt B. R. Farzin F. Jacquemont S. Greco C. M. Brunberg J. A.. (2004). Fragile-X-associated tremor/ataxia syndrome (FXTAS) in females with the FMR1 premutation. Am. J. Hum. Genet. 74, 1051–1056. 10.1086/420700, PMID: 15065016 PMC1181968

[ref29] Henderson J. M. Williams C. C. Falk R. J. (2005). Eye movements are functional during face learning. Mem. Cogn. 33, 98–106. 10.3758/BF03195300, PMID: 15915796

[ref30] Hernandez R. N. Feinberg R. L. Vaurio R. Passanante N. M. Thompson R. E. Kaufmann W. E. (2009). Autism spectrum disorder in fragile X syndrome: a longitudinal evaluation. Am. J. Med. Genet. A 149A, 1125–1137. 10.1002/ajmg.a.32848, PMID: 19441123 PMC2734278

[ref31] Hessl D. Rivera S. Koldewyn K. Cordeiro L. Adams J. Tassone F.. (2007). Amygdala dysfunction in men with the fragile X premutation. Brain 130, 404–416. 10.1093/brain/awl338, PMID: 17166860

[ref32] Hunter J. E. Allen E. G. Abramowitz A. Rusin M. Leslie M. Novak G. (2008). Investigation of phenotypes associated with mood and anxiety among male and female fragile X premutation carriers. Behav. Genet. 38, 493–502. 10.1007/s10519-008-9214-3. 18535897 PMC3696488

[ref33] Hurley R. S. Losh M. Parlier M. Reznick J. S. Piven J. (2007). The broad autism phenotype questionnaire. J. Autism Dev. Disord. 37, 1679–1690. 10.1007/s10803-006-0299-3, PMID: 17146701

[ref34] Klin A. Jones W. Schultz R. Volkmar F. Cohen D. (2002). Visual fixation patterns during viewing of naturalistic social situations as predictors of social competence in individuals with autism. Arch. Gen. Psychiatry 59, 809–816. 10.1001/archpsyc.59.9.809 12215080

[ref35] Klusek J. Fairchild A. J. Roberts J. E. (2018a). Vagal tone as a putative mechanism for pragmatic competence: an investigation of carriers of the FMR1 Premutation. J. Autism Dev. Disord. 49, 197–208. 10.1007/s10803-018-3714-7, PMID: 30097759 PMC6855249

[ref36] Klusek J. McGrath S. E. Abbeduto L. Roberts J. E. (2016). Pragmatic language features of mothers with the FMR1 premutation are associated with the language outcomes of adolescents and young adults with fragile X syndrome. J. Speech Lang. Hear. Res. 59, 49–61. 10.1044/2015_JSLHR-L-15-0102 26895548 PMC4867932

[ref37] Klusek J. Porter A. Abbeduto L. Adayev T. Tassone F. Mailick M.. (2018b). Curvilinear association between language disfluency and FMR1 CGG reapt size across the normal, intermediate, and premutation range. Front. Genet. 9:344. 10.3389/fgene.2018.00344, PMID: 30197656 PMC6118037

[ref38] Klusek J. Schmidt J. Fairchild A. J. Porter A. Roberts J. E. (2017). Altered sensitivity to social gaze in the *FMR1* premutation and pragmatic language competence. J. Neurodev. Disord. 9:31. 10.1186/s11689-017-9211-z, PMID: 28835209 PMC5569479

[ref39] LaFauci G. Adayev T. Kascsak R. Kascsak R. Nolin S. Mehta P. (2013). Fragile X screening by quantification of FMRP in dried blood spots by a luminex immunoassay. J. Mol. Diagn. 15, 508–517. 10.1016/j.jmoldx.2013.02.006. 23660422

[ref40] Landa R. Piven J. Wzorek M. M. Gayle J. O. Chase G. A. Folstein S. E. (1992). Social language use in parents of autistic individuals. Psychol. Med. 22, 245–254. 1574562 10.1017/s0033291700032918

[ref41] Lee M. Nayar K. Maltman N. Hamburger D. Martin G. E. Gordon P. C.. (2019). Understanding social communication differences in autism spectrum disorder and first-degree relatives: a study of looking and speaking. J. Autism Dev. Disord. 50, 2128–2141. 10.1007/s10803-019-03969-3, PMID: 30864059 PMC7261276

[ref42] Lord C. Rutter M. DeLavore P. C. Risi S. (2001). Autism diagnostic observation schedule. Los Angeles, CA: Western Psychological Services.

[ref43] Losh M. Adolphs R. Poe M. D. Couture S. Penn D. Baranek G. T.. (2009). Neuropsychological profile of autism and the broad autism phenotype. Arch. Gen. Psychiatry 66, 518–526. 10.1001/archgenpsychiatry.2009.34, PMID: 19414711 PMC2699548

[ref44] Losh M. Capps L. (2003). Narrative ability in high-functioning children with autism or Asperger's syndrome. J. Autism Dev. Disord. 33, 239–251. 10.1023/a:1024446215446, PMID: 12908827

[ref45] Losh M. Childress D. Lam K. Piven J. (2008). Defining key features of the broad autism phenotype: a comparison across parents of multiple- and single-incidence autism families. Am. J. Med. Genet. B Neuropsychiatr. Genet. 147B, 424–433. 10.1002/ajmg.b.30612 17948871 PMC2746421

[ref46] Losh M. Klusek J. Martin G. E. Sideris J. Parlier M. Piven J. (2012). Defining genetically meaningful language and personality traits in relatives of individuals with fragile X syndrome and relatives of individuals with autism. Am. J. Med. Genet. B Neuropsychiatr. Genet. 159B, 660–668. 10.1002/ajmg.b.32070, PMID: 22693142 PMC3740587

[ref47] Mailick M. R. Hong J. Rathouz P. Baker M. W. Greenberg J. S. Smith L.. (2014). Low-normal FMR1 CGG repeat length: phenotypic associations. Front. Genet. 5:309. 10.3389/fgene.2014.00309, PMID: 25250047 PMC4158814

[ref48] Mayer M. (1969). Frog, where are you? New York: Dial Press.

[ref49] Murray H. A. (1943). Thematic apperception test. Cambridge, Massachusetts: Harvard University Press.

[ref50] Nayar K. Gordon P. C. Martin G. E. Hogan A. L. La Valle C. McKinney W.. (2018). Links between looking and speaking in autism and first-degree relatives: insights into the expression of genetic liability to autism. Mol. Autism. 9:51. 10.1186/s13229-018-0233-5, PMID: 30338047 PMC6180594

[ref51] Nayar K. McKinney W. Hogan A. L. Martin G. E. La Valle C. Sharp K., et al. (2019). Language processing skills linked to FMR1 variation: a study of gaze-language coordination during rapid automatized naming among women with the FMR1 premutation. PLoS One 14:e0219924. 10.1371/journal.pone.0219924, PMID: 31348790 PMC6660192

[ref52] Norbury C. F. Bishop D. V. (2003). Narrative skills of children with communication impairments. Int. J. Lang. Commun. Disord. 38, 287–313. 10.1080/136820310000108133, PMID: 12851080

[ref53] Peters S. U. Beaudet A. L. Madduri N. Bacino C. A. (2004). Autism in Angelman syndrome: implications for autism research. Clin. Genet. 66, 530–536. 10.1111/j.1399-0004.2004.00362.x, PMID: 15521981

[ref80] Pierce K. Marinero S. Hazin R. McKenna B. Barnes C. C. Malige A. (2016). Eye tracking reveals abnormal visual preference for geometric images as an early biomarker of an autism spectrum disorder subtype associated with increased symptom severity. Biol. Psychiatry 79, 657–666. 10.1016/j.biopsych.2015.03.032, PMID: 25981170 PMC4600640

[ref54] Reilly J. Losh M. Bellugi U. Wulfeck B. (2004). “Frog, where are you?” narratives in children with specific language impairment, early focal brain injury, and Williams syndrome. Brain Lang. 88, 229–247. 10.1016/S0093-934X(03)00101-9, PMID: 14965544

[ref55] Sah W. -h. Torng P. -c. (2015). Narrative coherence of mandarin-speaking children with high-functioning autism spectrum disorder: an investigation into causal relations. First Lang. 35, 189–212. 10.1177/0142723715584227

[ref56] Schafer J. L. (1999). Multiple imputation: a primer. Stat. Methods Med. Res. 8, 3–15. 10.1177/096228029900800102 10347857

[ref57] Schneider A. Johnston C. Tassone F. Sansone S. Hagerman R. J. Ferrer E. (2016). Broad autism spectrum and obsessive-compulsive symptoms in adults with the fragile X premutation. Clin. Neuropsychol. 30, 929–943. 10.1080/13854046.2016.1189536. 27355445 PMC5004987

[ref58] Seltzer M. M. Baker M. W. Hong J. Maenner M. Greenberg J. Mandel D. (2012). Prevalence of CGG expansions of the FMR1 gene in a US population-based sample. Am. J. Med. Genet. B Neuropsychiatr. Genet. 159B, 589–597. 10.1002/ajmg.b.32065 22619118 PMC3391968

[ref59] Shelton A. L. Cornish K. M. Godler D. E. Clough M. Kraan C. Bui M. (2015). Delineation of the working memory profile in female FMR1 premutation carriers: the effect of cognitive load on ocular motor responses. Behav. Brain Res. 282, 194–200. 10.1016/j.bbr.2015.01.011. 25591477

[ref60] Shelton A. L. Cornish K. Kraan C. Georgiou-Karistianis N. Metcalfe S. A. Bradshaw J. L.. (2014). Exploring inhibitory deficits in female premutation carriers of fragile X syndrome: through eye movements. Brain Cogn. 85, 201–208. 10.1016/j.bandc.2013.12.006, PMID: 24424424

[ref61] Shelton A. L. Cornish K. M. Kraan C. M. Lozano R. Bui M. Fielding J. (2016). Executive dysfunction in female FMR1 Premutation carriers. Cerebellum 15, 565–569. 10.1007/s12311-016-0782-0, PMID: 27126308

[ref62] Shic F. Bradshaw J. Klin A. Scassellati B. Chawarska K. (2011). Limited activity monitoring in toddlers with autism spectrum disorder. Brain Res. 1380, 246–254. 10.1016/j.brainres.2010.11.074, PMID: 21129365 PMC3050079

[ref63] Steinberg J. Webber C. (2013). The roles of FMRP-regulated genes in autism spectrum disorder: single- and multiple-hit genetic etiologies. Am. J. Hum. Genet. 93, 825–839. 10.1016/j.ajhg.2013.09.013 24207117 PMC3824119

[ref64] Stuart E. A. Azur M. Frangakis C. Leaf P. (2009). Multiple imputation with large data sets: a case study of the children's mental health initiative. Am. J. Epidemiol. 169, 1133–1139. 10.1093/aje/kwp026 19318618 PMC2727238

[ref65] Tager-Flusberg H. (1995). ‘Once upon a ribbit’: stories narrated by autistic children. Br. J. Dev. Psychol. 13, 45–59. 10.1111/j.2044-835X.1995.tb00663.x

[ref66] Thao L. T. P. Geskus R. (2019). A comparison of model selection methods for prediction in the presence of multiply imputed data. Biom. J. 61, 343–356. 10.1002/bimj.201700232, PMID: 30353591 PMC6492211

[ref67] Thurber C. Tager-Flusberg H. (1993). Pauses in the narratives produced by autistic, mentally retarded, and normal children as an index of cognitive demand. J. Autism Dev. Disord. 23, 309–322. 8331049 10.1007/BF01046222

[ref68] Tottenham N. Tanaka J. W. Leon A. C. McCarry T. Nurse M. Hare T. A. (2009). The NimStim set of facial expressions: judgments from untrained research participants. Psychiatry Res. 168, 242–249. 10.1016/j.psychres.2008.05.006. 19564050 PMC3474329

[ref69] van Buuren S. Groothuis-Oudshoorn K. (2011). Mice: multivariate imputation by chained equations in R. J. Stat. Softw. 45, 1–67. 10.18637/jss.v045.i03

[ref70] Vink G. Frank L. E. Pannekoek J. Van Buuren S. (2014). Predictive mean matching imputation of semicontinuous variables. Statistica Neerlandica 68, 61–90. 10.1111/stan.12023

[ref71] von Hippel P. T. (2020). How many imputations do you need? A two-stage calculation using a quadratic rule. Sociol. Methods Res. 49, 699–718. 10.1177/0049124117747303 PMC11361408 39211325

[ref72] Wang J. Y. Hessl D. Iwahashi C. Cheung K. Schneider A. Hagerman R. J. (2013). Influence of the fragile X mental retardation (FMR1) gene on the brain and working memory in men with normal FMR1 alleles. NeuroImage 65, 288–298. 10.1016/j.neuroimage.2012.09.075. 23063447 PMC3540208

[ref73] Wechsler D. (1999). WASI: Wechsler abbreviated scale of intelligence. San Antonio, TX: Psychological Corporation.

[ref74] Weiler I. J. Greenough W. T. (1999). Synaptic synthesis of the fragile X protein: possible involvement in synapse maturation and elimination. Am. J. Med. Genet. 83, 248–252. 10208156 10.1002/(sici)1096-8628(19990402)83:4<248::aid-ajmg3>3.0.co;2-1

[ref75] Wheeler A. C. Bailey D. B. Jr. Berry-Kravis E. Greenberg J. Losh M. Mailick M. (2014). Associated features in females with an FMR1 premutation. J. Neurodev. Disord. 6:30. 10.1186/1866-1955-6-30. 25097672 PMC4121434

[ref76] Winston M. Nayar K. Hogan A. L. Barstein J. La Valle C. Sharp K.. (2020). Physiological regulation and social-emotional processing in female carriers of the FMR1 premutation. Physiol. Behav. 214:112746. 10.1016/j.physbeh.2019.112746, PMID: 31765665 PMC6992413

[ref77] Yucel G. H. Belger A. Bizzell J. Parlier M. Adolphs R. Piven J. (2015). Abnormal neural activation to faces in the parents of children with autism. Cereb. Cortex 25, 4653–4666. 10.1093/cercor/bhu147, PMID: 25056573 PMC4635912

